# Basic and clinical immunology – 3013. Resource limited estimation of HIV-1 viral load from CD4 cell count using coupled bacterial foraging/jumping frogs algorithm

**DOI:** 10.1186/1939-4551-6-S1-P189

**Published:** 2013-04-23

**Authors:** Kamalanand Krishnamurthy, Mannar Jawahar Ponnuswamy

**Affiliations:** 1 Department of Instrumentation Engineering, MIT Campus, Anna University, Chennai, India; 2 Centre for University Industry Collaboration, Anna University, Chennai, India

## Background

Viral load (VL) testing is highly important for detection of antiretroviral treatment (ART) failure in AIDS patients. However, due to the cost and availability of viral load testing instruments, VL testing is not being performed in many developing and underdeveloped countries. Such countries depend on CD4 tests for staging the HIV infection and for initiating ART, since CD4 testing is simple and less expensive. The objective of this work is to estimate the HIV-1 viral load from CD4 cell count using an hybrid artificial intelligence technique known as the coupled bacterial foraging/jumping frogs optimization algorithm and the three dimensional mathematical model of HIV infection.

## Methods

In this work, a method for estimating the HIV-1 viral load from CD4 cell counts, using a hybrid artificial intelligence technique is proposed. The coupled bacterial foraging/jumping frogs optimization procedure is a mathematical procedure which combines the decision making capabilities of the *E.Coli* bacteria and frogs. The coordinates of a bacterium and a frog represents an individual solution of the optimization problem. The final solution is obtained as the set of trial solutions converges towards the optimal solution. The computer program for estimation was developed using Matlab 7.10.1 running on a system with 1.73 GHz processor.

## Results

The accuracy in estimation of HIV-1 viral load using the proposed method was found to be 94.58%. Further, the estimation error in the acute stage of the disease was found to be 4.29%, whereas, the estimation error in the chronic phase of the disease was found to be 0.83%. Also, the average correlation value between the actual and estimated HIV-1 viral load was found to be 0.99. The total time for estimation of HIV-1 viral load was 15.22 seconds.

## Conclusions

The proposed approach for HIV-1 viral load estimation appears to be efficient and useful as a low cost strategy for VL testing in resource limited settings.

